# Metabolic Abnormalities of Chronic High-Dose Glucocorticoids Are Not Mediated by Hypothalamic AgRP in Male Mice

**DOI:** 10.1210/en.2019-00018

**Published:** 2019-02-22

**Authors:** Charlotte Sefton, Alison Davies, Tiffany-Jayne Allen, Jonathan R Wray, Rosemary Shoop, Antony Adamson, Neil Humphreys, Anthony P Coll, Anne White, Erika Harno

**Affiliations:** 1 Division of Diabetes, Endocrinology and Gastroenterology, Faculty of Biology, Medicine and Health, University of Manchester, Manchester Academic Health Sciences Centre, Manchester, United Kingdom; 2 Manchester Transgenic Unit, University of Manchester, Manchester Academic Health Sciences Centre, Manchester, United Kingdom; 3 University of Cambridge Metabolic Research Laboratories and MRC Metabolic Diseases Unit, Wellcome-MRC Institute of Metabolic Science, Addenbrooke’s Hospital, Cambridge, United Kingdom

## Abstract

Glucocorticoids are potent and widely used medicines but often cause metabolic side effects. A murine model of corticosterone treatment resulted in increased hypothalamic expression of the melanocortin antagonist AgRP in parallel with obesity and hyperglycemia. We investigated how these adverse effects develop over time, with particular emphasis on hypothalamic involvement. Wild-type and *Agrp^−/−^* male mice were treated with corticosterone for 3 weeks. Phenotypic, biochemical, protein, and mRNA analyses were undertaken on central and peripheral tissues, including white and brown adipose tissue, liver, and muscle, to determine the metabolic consequences. Corticosterone treatment induced hyperphagia within 1 day in wild-type mice, which persisted for 3 weeks. Despite this early increase in food intake, the body weight only started to increase after 10 days. Hyperinsulinemia occurred at day 1. Also, although after 2 days, alterations were present in the genes often associated with insulin resistance in several peripheral tissues, hyperglycemia only developed at 3 weeks. Throughout, sustained elevation in hypothalamic *Agrp* expression was present. Mice with *Agrp* deleted [using clustered regularly interspaced short palindromic repeats (CRISPR)–Cas9, *Agrp*^−/−^] were partially protected against corticosterone-induced hyperphagia. However, *Agrp*^−/−^ mice still had corticosterone-induced increases in body weight and adiposity similar to those of the *Agrp*^+/+^ mice. Loss of *Agrp* did not diminish corticosterone-induced hyperinsulinemia or correct changes in hepatic gluconeogenic genes. Chronic glucocorticoid treatment in mice mimics many of the metabolic side effects seen in patients and leads to a robust increase in *Agrp*. However, AgRP does not appear to be responsible for most of the glucocorticoid-induced adverse metabolic effects.

Synthetic glucocorticoids are a widely used class of medicines, important in the treatment of numerous inflammatory disorders, including rheumatoid arthritis and asthma, and regularly used after organ transplantation. In the short term, they have generally been well tolerated; however, longer term administration can lead to a range of adverse effects, including weight gain, hyperglycemia, and osteoporosis. Although prophylactic treatment will often be used to ameliorate the skeletal side effects such as osteoporosis, no specific interventions are available to mitigate the metabolic changes.

The mechanisms by which chronically increased glucocorticoids result in these adverse metabolic profiles are complex. The increased incidence of obesity seen in patients with chronically elevated glucocorticoids might derive from glucocorticoid-induced hyperphagia. This has been seen in several rodent studies of chronic glucocorticoid treatment (1–3). The hypothalamus has a key role in appetitive behavior, and glucocorticoids might drive this change in food intake by acting within this brain region. This has been evidenced from the glucocorticoid effects on a number of neuropeptides, including POMC (4, 5), AgRP (6, 7), and NPY (4, 6, 8). However, the reported effects of glucocorticoids on these energy-regulatory neuropeptides have often been conflicting.

The actions of glucocorticoids on peripheral tissues are complex. For example, in white adipose tissue (WAT), glucocorticoids are known to be lipolytic (9), but can also cause fat redistribution, typically diminishing subcutaneous fat and increasing intra-abdominal obesity (10). Glucocorticoids can also affect brown adipose tissue (BAT), with a reduction in the levels of UCP-1, an essential protein in nonshivering thermogenesis, reported in multiple rodent models (11, 12). However, species-specific differences in this response appear to be present (13), and whether this potential suppression of BAT activity results in changes in energy expenditure sufficient to alter body fat stores in the longer term remains to be fully determined. Similarly, although molecular mechanisms for the acute effects of glucocorticoids on hepatic glucose output have been well-described (14), it is less clear what effects chronic glucocorticoid administration might have in the presence of prolonged hyperinsulinemia and insulin resistance.

We have previously reported that mice administered corticosterone (75 µg/mL) in drinking water had increased hypothalamic corticosterone levels, which caused changes in the glucocorticoid target genes and an elevation in the orexigenic neuropeptide AgRP after 4 weeks (3). Increasing evidence has shown that central molecular pathways involving neuropeptides such as AgRP are able to signal from the hypothalamus to have specific metabolic effects on peripheral tissues, including WAT (15), BAT (16), and liver (17). However, the evolution and the mechanisms driving glucocorticoid-induced metabolic effects remain unclear. Therefore, in the present study we investigated how and when the different adverse effects of glucocorticoids develop over time and used a mouse model of *Agrp* deficiency to determine the role of AgRP in their development. We found an immediate increase in food intake but a delayed increase in body weight and fat pad mass. Furthermore, although *Agrp* increased rapidly in parallel with elevated food intake, loss of *Agrp* did not protect from weight gain or hyperinsulinemia.

## Materials and Methods

### Generation of *Agrp*^−/−^ mice

**Figure 1.**
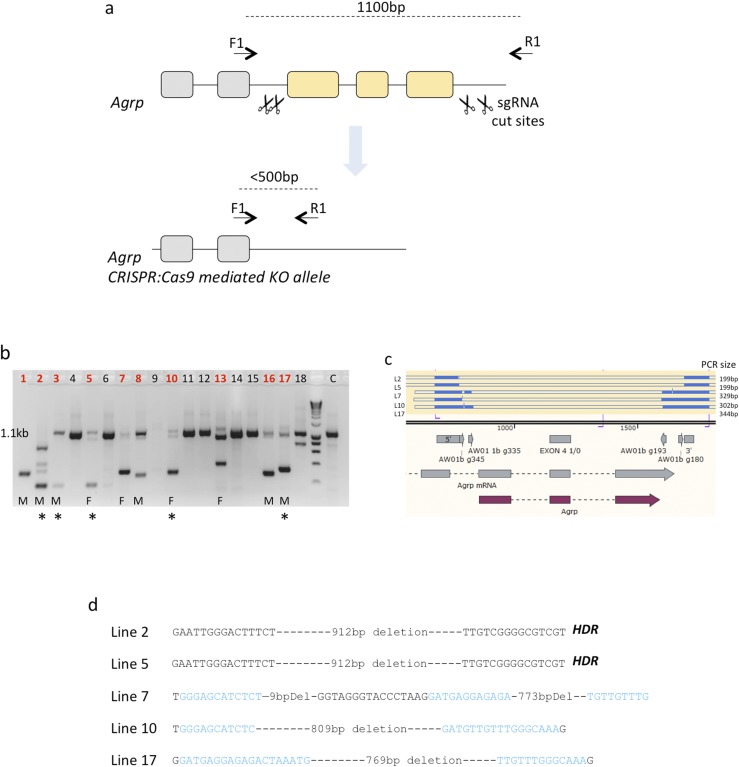
(a) Schematic representation of *Agrp* gene deletion by CRISPR-Cas9. (b) Genotyping of founders. Most pups displayed evidence of some cut and repair activity, with 10 of 18 showing a large fragment deletion (indicated by red numbering). Asterisks indicated mice taken forward for full sequencing. (c) Summary of alignments of products against wild-type sequence. Blue bars indicate alignment; white bars, loss of alignment/deletion. (d) Sequencing of founder lines, with specific deletions indicated. Lines 2 and 5 are perfect homology-directed repair (HDR); lines 7 and 17 have deletions of DNA between g335 and g193; and line 10 has deletion between g335 and g193.

To generate an *Agrp* knockout transgenic mouse line (*Agrp*^−/−^) we used clustered regularly interspaced short palindromic repeats (CRISPR)-Cas9 technology, and designed four single-guide RNA (sgRNA), which flanked the entire protein coding sequences (Fig. 1). Two sgRNA were targeted upstream and two were targeted downstream of the gene, which, in the event of combined cutting, would result in excision of the full gene. We also designed a single-stranded DNA template to facilitate a precise homology-directed repair event. The sgRNA were selected using the Sanger WTSI website [available at: http://www.sanger.ac.uk/htgt/wge/ (18)] and stringent criteria for off-target predictions. Guides with mismatch of 0, 1, or 2 for elsewhere in the genome were discounted. Guides with mismatch 3 were tolerated if the predicted off targets were not exonic.

We synthesized sgRNA using previously described protocols (19). In brief, a forward oligonucleotide for each sgRNA was designed according to the template from CRISPR_F (replacing GGN18-20 with the guide sequence), and 10 µM of forward primer combined with 10 µM of universal CRISPRsgRNA primer were used in a PCR reaction with high-fidelity polymerase (Phusion; New England BioLabs, Ipswich, MA). Next, 200 ng of the resulting amplicon was used as a template in an *in vitro* transcription reaction (HiScribe; New England BioLabs, Hitchen, UK), before purification (MEGAclear; Ambion, Paisley, UK) and quantification by NanoDrop (Thermo Fisher Scientific/Life Sciences, Paisley, UK).

An injection mix of the four sgRNA (20 ng/µL each) and Cas9 mRNA (100 ng/µL) was prepared and directly microinjected into B6D2F1 (Envigo, Huntington, UK) zygote pronuclei using standard protocols. The zygotes were cultured overnight, and the resulting two cell embryos were surgically implanted into the oviduct of day 0.5 post-coitum pseudopregnant CD1 mice. Potential founder mice were screened by PCR, with genotyping primers F1:ctgccatataagctcagggca and R1:tggtgccttaaactcgccc. On a wild-type template, these primers will amplify an 1100-bp band. In contrast, excision of the gene results in band of ∼200 to 300 bp. We obtained several candidate mice (10 of 18) and took 5 forward for sequencing after PCR-blunt cloning. We confirmed the full loss of the gene in each. Two founder mice (mice 2 and 5; Fig. 1) were identified and then back-crossed to C57Bl/6J wild-type mice to assess germline penetrance, with line 5 taken forward. The colonies were genotyped as described. The *Agrp*^−/−^ mice produced offspring litters in Mendelian ratios and were maintained by intercrosses on the mixed C57Bl/6J and DBA/2J background.

### Study design

C57Bl/6J mice were purchased from Charles River (Margate, Kent, UK), and *Agrp*^−/−^ mice and their *Agrp*^+/+^ littermates were bred at the University of Manchester. All mice were maintained in a 12:12 light/dark cycle (lights on at 7:00 am and lights off at 7:00 pm) in specific pathogen-free cages with wood chip bedding and environmental enrichment. Food and water were available *ad libitum*.

Ten-week-old male mice were housed singly and acclimatized for 1 week, before a 2-week baseline, during which the body weight and food and water intake were monitored daily (short studies) or twice weekly (3-week studies). The mice were assigned to treatment groups of corticosterone (Sigma-Aldrich, Gillingham, Dorset, UK; 75 µg/mL in 1% ethanol) (3) or vehicle (1% ethanol) in drinking water, such that each group had mice with equivalent baseline body weight and food intake. *Agrp*^−/−^ and *Agrp*^+/+^ mice were randomly assigned at genotyping. The dose of corticosterone was chosen based on a previous dose-finding study, in which lower doses of corticosterone were found to not give a full range of metabolic phenotypic effects. The mice were treated for 1, 2, or 3 days or 3 weeks, and their body weight and food and water intake were measured just as during the baseline period. On veterinary advice, mice drinking >15 mL/d during treatment were changed to water during the day. This occurred for ∼20% of the mice administered corticosterone for 3 weeks. At termination, the mice were tail bled to measure the glucose and insulin before euthanasia by increasing carbon dioxide levels, followed by exsanguination. The tissues were weighed and then snap frozen (Qiagen, Manchester, UK) or placed in 10% formalin. Whole hypothalami were isolated for mRNA expression analysis. Microdissection scissors were used to cut immediately caudal to the optic chiasm and dorsally by the mammillothalamic tract. The dissection was limited laterally by the hypothalamic sulci. The entire hypothalamus was then removed and placed in RNAlater (Qiagen) before RNA extraction.

All experiments were performed in accordance with the UK Home Office legislation [Animal (Scientific Procedures) Act 1986] and were approved by the University of Manchester local ethics committee. All experiments were performed in accordance with the relevant guidelines and regulations.

### Biochemical measurements

Glucose was measured fresh in tail-prick blood samples using a glucometer (Accu-Chek; Roche, West Sussex, UK). For other analytes, the blood was centrifuged and plasma removed and stored at −80°C. Leptin (Millipore, Watford, UK) and insulin (CrystalChem, Elk Grove Village, IL) were measured using an ELISA. For the homeostatic model assessment for insulin resistance, the mice were fasted for 16 hours overnight before samples were taken (20).

### Real-time quantitative PCR

RNA was extracted using an RNeasy mini kit (Qiagen) with on-column genomic DNA digestion according to the manufacturer’s protocol, with an additional phenol extraction for adipose tissue. RNA was reverse transcribed (High Capacity cDNA RT; Applied Biosystems, Paisley, UK), and transcript levels were determined using a Prism 7900HT (Applied Biosystems) with either TaqMan Gene Expression Assays and TaqMan Universal Master Mix II (Applied Biosystems) or SYBR assays designed with primer-BLAST software (National Center for Biotechnology Information, Bethesda, MD) and GoTaq qPCR Master Mix (Promega, Southampton, UK). Catalogue numbers and primer sequences are listed in Table 1. Samples were quantified using a standard curve with HPRT or TBP for TaqMan or SYBR assays, respectively, as housekeepers, and the vehicle group as calibrator.

**Table 1.** Sequence Information or Reference Numbers of Real-Time Quantitative PCR Assays Used

Gene	Reference Number	Forward 5′-3′	Reverse 5′-3′
*Agrp*	Mm00475829_g1		
*Avp*	Mm01271704_m1		
*Bdnf*		GCTCCGGGTTGGTATACTGG	CACCTGGTGGAACTTCTTTGC
*Cartpt*	Mm04210469_m		
*Cidea*	Mm00432554_m1		
*Crh*	Mm01293920_s1		
*Fkbp5*		AGCAACGGTAAAAGTCCACCT	TTCCCCAACAACGAACACCA
*G6pc*		GTGAGACCGGACCAGGAAGTC	ATCCCAACCACAAGATGACGTT
*Gad1*	Mm04207432_g1		
*Gad2*	Mm00484623_m1		
*Gal*		GCCCACATGCCATTGACAAC	GCACATCAACACTTCCTGGTC
*Gck*		AAGCTGCACCCGAGCTTCAA	GCTGCCCTCCTCTGATTCAA
*Ghsr*		AGATCGCGCAGATCAGTCAG	GTATTGATGCTCGACTTTGTCCA
*Hcrt*		TTTGGACCACTGCACTGAAGA	GGCCCAGGGAACCTTTGTAGA
*Hprt*	Mm03024075_m1		
*Hsd11b1*	Mm00476182_m1		
*Irs1*		CAAGACGCTCCAGTGAGGATT	TTTAGGTCTTCATTCTGCTGTGA
*Klf4*		AGAACAGCCACCCACACTTG	GTGGTAAGGTTTCTCGCCTGT
*Lep*		GCTCCAGAAGAAGAGGACCAA	GACTGAATTTCCAAAAGCCTGAA
*Mc3r*		TCAAGGAGATTCTCTGCGGC	ACACCCTTTTACGTCCCGTC
*Mc4r*		GGGTCGGAAACCATCGTCAT	CTGCAAATGGATGCGAGCAA
*Nmb*		CCTGCTCTTCGCATTGTTCG	AAGTGACCGGTCGCCCA
*Npy*		ATGCTAGGTAACAAGCGAATGG	TGTCGCAGAGCGGAGTAGTAT
*Nr3c1*		AGCTCCCCCTGGTAGAGAC	GGTGAAGACGCAGAAACCTTG
*PCK1*		CCTAGTGCCTGTGGGAAGAC	AAGTTGCCTTGGGCATCAAAC
*Pomc*		ATGCCGAGATTCTGCTACAGT	TCCAGCGAGAGGTCGAGTTT
*Ppargc1a*		AGCCGTGACCACTGACAACGAG	GCTGCATGGTTCTGAGTGCTAAG
*Prdm16*		GACATTCCAATCCCACCAGA	CACCTCTGTATCCGTCAGCA
*Pygl*		CCACTCGGACATCGTGAAGA	CCAATTTTCTCCGCTATCAAGTC
*Sim1*		GAGGAGGGAGAAAGAAAACAGTG	CCAAGCCCTTCTGGAAAGACC
*Sla2a2*		GATCGCTCCAACCACACTCA	CTGAGGCCAGCAATCTGACTA
*Slc17a6*		GGCTGGACACCAGTCTTTACAA	TTCTTCAGCACCCTGTAGATCTGT
*Slc32a1*		GGGTCACGACAAACCCAAGA	GCACGAACATGCCCTGAATG
*Tbp*		GGGAGAATCATGGACCAGAA	GATGGGAATTCCAGGAGTCA
*Trh*		CCTTGGTGCTGCCTTAGATTCC	CCCTCTCTTCGGCTTCAACG
*Tsc22d3*		GCAGGCCATGGACCTCGTGAAG	TCAGGAGGGTGTTCTCGCGCT
*Ucp1*		ACTGCCACACCTCCAGTCATT	CTTTGCCTCACTCAGG ATTGG
*p110b*		GCGCGGGGCAGTTCATCTTCTAA	GAGGCATGATAGGGCGGAAGCA
*p85α*		GCCAAGGAAACTGTCGCACACA	GGGGCAGTGCTGGTGGATCCAT

### Immunohistochemistry

Tissues were fixed in 10% formalin for 24 hours, cryoprotected in 30% sucrose for 24 hours, frozen in OCT (Klinipath, Gelderland, Netherlands) using supercooled 2-methylbutane, and stored at −80°C. All tissues were cryosectioned at 12 µm.

### Hematoxylin and eosin staining

Frozen sections were rehydrated in a graded ethanol series (from 100% to 70%) and stained with Shandon Gills no. 2 hematoxylin and Shandon eosin Y (both Thermo Fisher Scientific/Life Sciences), before dehydration in increasing ethanol concentrations (70% to 100%). Sections were mounted in DPX medium (Sigma-Aldrich) and cover slipped. The images presented are representative of three sections per mouse and four mice per group.

### Oil Red O staining

Sections were stained with Oil Red O (CI 26125, Sigma-Aldrich) (21), and counterstained with Shandon Gills no. 2 hematoxylin (Thermo Fisher Scientific/Life Sciences).

### *Agrp in situ* hybridization

*In situ* hybridization was performed on frozen brain sections, three per group (*Agrp*^−/−^ and *Agrp*^+/+^), as previously described (3). An antisense digoxigenin-labeled riboprobe was generated using a T7 *in vitro* transcription system (Promega) in the presence of a digoxigenin labeling mix (Roche) and an RNA expression vector pGM-5ZF(+) vector (Promega) containing the *Agrp* cDNA sequence from the 196-777 bp of sequence NM_001271806.1_ (581 bp).

The riboprobe was hybridized overnight at 60°C. Next, the sections were rinsed in 4× SSC (Promega), followed by 2 µg/mL RNaseA (Sigma-Aldrich) in 10 mM Tris (pH 8.0), 1 mM EDTA, and 0.5 M NaCl. After a final wash in 0.5× SSC at 60°C, the sections were blocked in 2% fetal calf serum and incubated with anti-digoxigenin-AP antibody (1:1500; Roche) (22) overnight at 4°C. After washing, the signal was revealed by incubation in 2% NBT/BCIP solution (Roche) for 2 hours.

### Imaging studies

All images were visualized using a 20×/0.80 Plan Apo objective using the 3D-Histech Pannoramic-250 Flash II slide-scanner (3D-Histech, Budapest, Hungary). Snapshots of the slide-scans were taken using CaseViewer software (3D-Histech).

### Western blot

BAT was thawed in RIPA buffer (Sigma-Aldrich) supplemented with EDTA and protease inhibitors (Stratech, Ely, UK) before being homogenized (Qiagen). The protein concentration was estimated using the Bradford assay (Bio-Rad, Watford, UK). The proteins were separated using SDS-PAGE (Thermo Fisher Scientific/Life Sciences) and transferred to polyvinylidene difluoride membrane. The membranes were blocked in Odyssey-PBS Blocking buffer (LI-COR Biosciences, Cambridge, UK) for 1 hour before incubating with primary antibodies [1:5000 anti-*β*-actin; Proteintech, UK; 60008-1-Ig (23); 1:1000 anti-UCP-1; Abcam, UK; ab10983 (24)] diluted in Tris-buffered saline-Tween 20 (TBS-T), overnight at 4°C. After washing in TBS-T, the membranes were incubated with secondary antibodies [1:5000 800CW donkey anti-rabbit; 926-32213 (25); or 680RD goat anti-mouse; 925-68070 (26); both LI-COR Biosciences] prepared in TBS-T for 1 hour at room temperature. The membranes were visualized using an Odyssey CLx scanner and protein expression was determined using Image Studio^TM^ Lite, version 5.2, software (LI-COR Biosciences) with *β*-actin as the loading control.

### Indirect calorimetry

A comprehensive laboratory animal monitoring system (Columbus Instruments, Columbus, OH) was used to measure the metabolic gases, every 18 minutes, in a separate cohort of mice. The mice were acclimatized to cages for 24 hours, after which oxygen consumption, carbon dioxide production, and respiratory exchange ratio (RER) were measured for a further 48 hours. The mice were then treated for a further 14 days. Energy expenditure was analyzed using analysis of covariance (ANCOVA) using body weight as a covariate (27) and as hourly averages normalized to body weight. The RER data were plotted as hourly averages and normalized to body weight. The mice kept in the comprehensive laboratory animal monitoring system cages showed phenotypic changes with corticosterone treatment identical to those of the mice kept in home cages.

### Statistical analysis

The data were analyzed using Prism, version 7.0 (GraphPad, San Diego, CA) and are presented as mean ± SEM. For normally distributed data, Student *t* test was performed between two groups. For real-time quantitative PCR data, the groups were compared using a Mann-Whitney *U* test. A two-way ANOVA was used for body weight and food intake data. ANCOVA was performed using SPSS (IBM Corp., Armonk, NY). *P* values of 0.05 were considered to indicate statistical significance.

## Results

### Excess glucocorticoids rapidly increase food intake, with a delayed gain in body weight and fat pad mass

**Figure 2.**
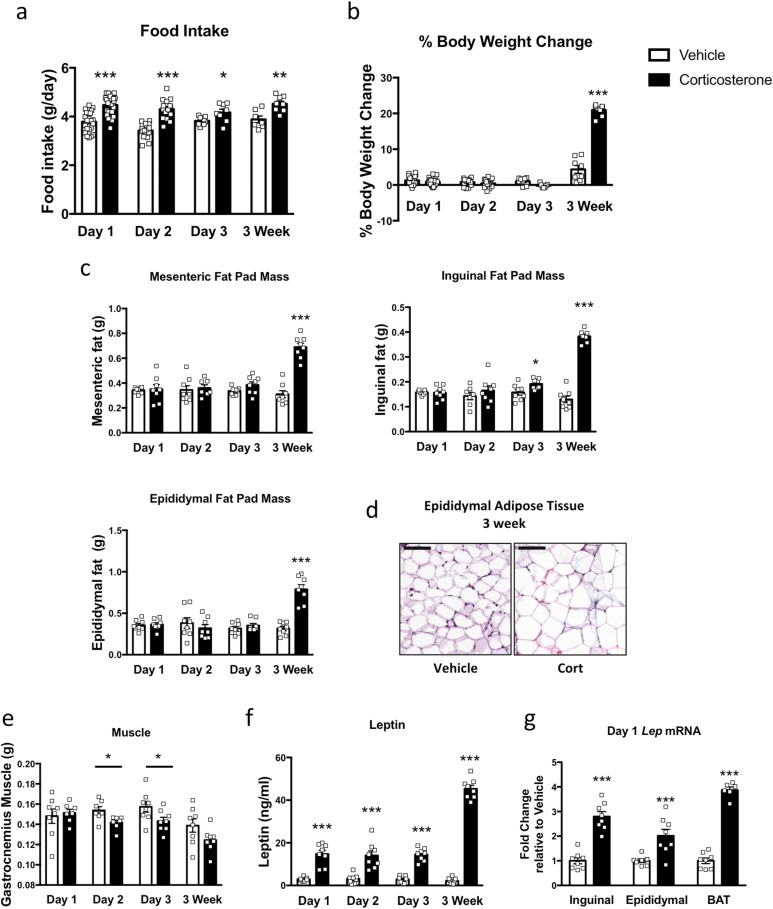
Food intake and leptin increased with 1 day of treatment, but body weight and fat pad mass increased later. (a) Food intake was increased after 1, 2, and 3 days and 3 weeks of corticosterone treatment (day 1, n = 23 to 24; day 2, n = 15; day 3, n = 8; 3 weeks, n = 7 to 8). (b) Percentage body weight change (day 1, n = 23; day 2, n = 15; day 3; n = 8; 3 weeks, n = 7 to 8). (c) Mesenteric, inguinal, and epididymal fat pad mass were increased after 3 weeks of corticosterone treatment (n = 7 to 8). (d) Hematoxylin and eosin staining of epididymal adipose tissue demonstrated larger adipocyte size after 3 weeks of corticosterone treatment. Scale bar, 100 µm (n = 4; three sections per slide). (e) Gastrocnemius muscle weight after 1, 2, and 3 days and 3 weeks of corticosterone treatment (n = 7 to 8). (f) Circulating leptin was increased after 1 day and had increased further after 3 weeks of corticosterone treatment (n = 6 to 8). (g) *Lep* mRNA expression was increased after 1 day of corticosterone treatment (n = 6 to 8). White bars indicate vehicle; black bars, 75 µg/mL corticosterone treatment. *P < 0.05; **P < 0.01; ***P < 0.001. (a–e) Unpaired Student *t* test; (f) two-way ANOVA with Fisher least squares difference post-test; and (g) unpaired Mann-Whitney *t* test.

Food intake increased by 18% after 1 day of corticosterone treatment and was still elevated at 3 weeks (Fig. 2a), with mice ingesting nearly 20% more each day. However, we did not observe a change in body weight during the first 3 days of corticosterone treatment (Fig. 2b). Overall, the mice’s body weight did not start to differ until day 10 (data not shown) and was substantially increased by 3 weeks (Fig. 2b).

A small increase was found in inguinal fat on day 3; however, by 3 weeks, inguinal, epididymal, and mesenteric fat pads had all at least doubled in mass (Fig. 2c). This was accompanied by an increase in adipocyte size as shown in the epididymal fat (Fig. 2d). Additionally, the gastrocnemius muscle weight had decreased after days 2 and 3. We also found a trend toward a decrease in the mass of this tissue at 3 weeks (Fig. 2e).

Circulating leptin levels had increased fivefold after day 1 of corticosterone treatment (Fig. 2f), accompanied by at least a twofold increase in *Lep* mRNA in WAT and BAT (Fig. 2g). At 3 weeks, the leptin concentrations had increased 65-fold in the plasma (Fig. 2f), with a greater fold increase in *Lep* mRNA expression in inguinal (11.2-fold) and epididymal (4.8-fold) adipose tissue and in BAT (10.3-fold; data not shown).

### Corticosterone increases BAT mass with no change in energy expenditure

**Figure 3.**
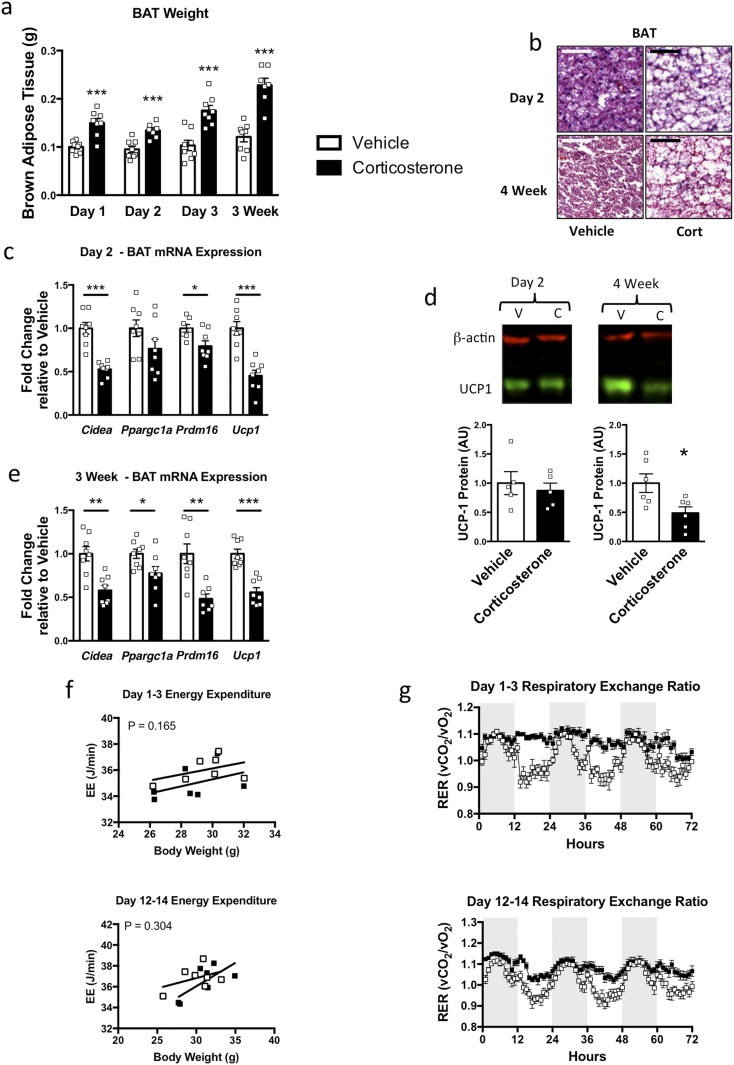
Effect of corticosterone treatment on BAT mass and RER. (a) BAT mass was increased from day 1 of corticosterone treatment (n = 7 to 8). (b) Representative image of hematoxylin and eosin staining of BAT after 2 days and 4 weeks of corticosterone treatment. Scale bar, 100 µm (n = 4; three sections per slide). (c) Decreased thermogenic genes after 2 days of corticosterone treatment (n = 7 to 8). (d) UCP-1 protein expression was unchanged after 2 days and had decreased after 4 weeks of corticosterone treatment (n = 5 to 6). (e) Decreased thermogenic genes in BAT after 3 weeks of corticosterone treatment (n = 7 to 8). (f) ANCOVA analysis showing no change in energy expenditure on days 1 to 3 or days 12 to 14 of corticosterone treatment (cohort 2; n = 7). (g) RER was increased during the day on days 1 to 3 and days 12 to 14 of corticosterone treatment in cohort 2 (n = 7). White bars indicate vehicle; black bars, 75 µg/mL corticosterone treatment. *P < 0.05; **P < 0.01; ***P < 0.001. (a, d) Unpaired Student *t* test; (c, e) unpaired Mann-Whitney *t* test; (f) ANCOVA analysis; and (g) two-way ANOVA with Fisher least squares difference post-test.

The BAT mass was increased with 1 day of corticosterone treatment, which was maintained over the other evaluation points (Fig. 3a). This was associated with lipid infiltration into the tissue at 2 days (Fig. 3b). Additionally, there were changes in genes associated with thermogenesis in BAT after 2 days of corticosterone treatment. *Cidea*, *Prdm16*, and *Ucp1* mRNA expression were decreased (Fig. 3c). Also, at 3 weeks, *Ppargc1a* mRNA expression had decreased (Fig. 3e). UCP-1 protein levels were not reduced until 4 weeks (Fig. 3d), although the lipid infiltration observed at day 2 remained (Fig. 3b).

The energy expenditure of the mice was not changed by corticosterone treatment during either the light or dark phase during days 1 to 3 or days 12 to 14 (data not shown). Also, no substantial effect of corticosterone was found on energy expenditure after ANCOVA with body weight as a covariate (Fig. 3f). However, in the corticosterone-treated group, a sustained elevation in RER during the light phase was found, in contrast to the decrease in RER seen during the light phase in the vehicle group (Fig. 3g).

### Corticosterone treatment altered markers of insulin resistance

**Figure 4.**
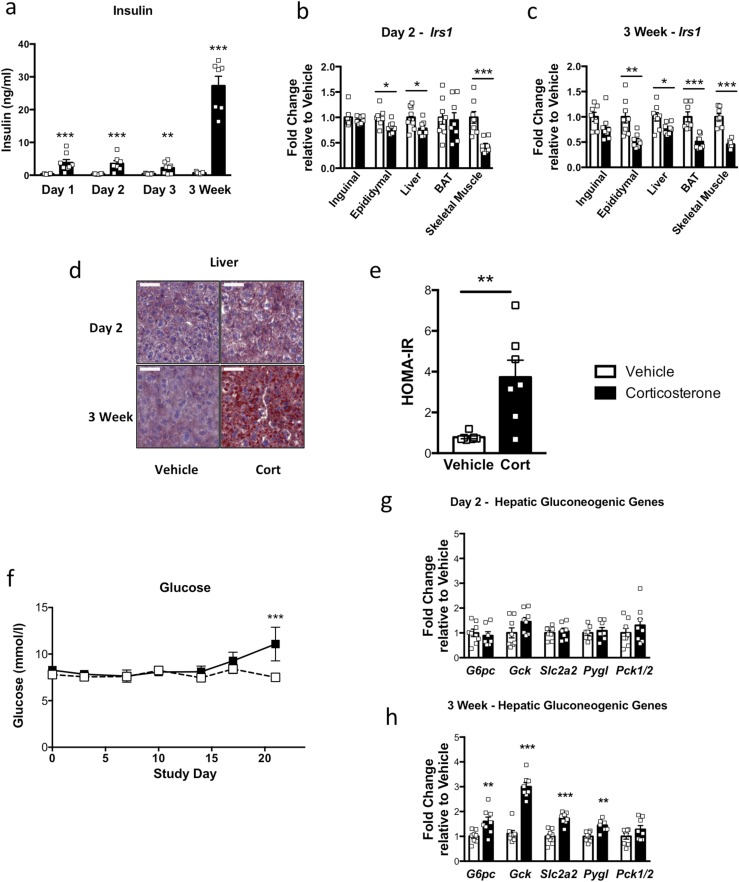
Effect of corticosterone treatment on hyperglycemia and insulin resistance. (a) Circulating insulin was increased after 1 day and was further increased after 3 weeks of corticosterone treatment (n = 6 to 8). (b) Corticosterone decreased epididymal adipose tissue, liver, and skeletal muscle *Irs1* after 2 days of corticosterone (n = 7 to 8). (c) Epididymal adipose, liver, BAT, and skeletal muscle *Irs1* had decreased after 3 weeks of corticosterone treatment (n = 7 to 8). (d) Oil red O staining in liver after 2 days and 3 weeks of corticosterone treatment. Scale bar, 50 µm (n = 4; three sections per mouse). (e) Homeostatic model assessment for insulin resistance was increased after 3 weeks of corticosterone treatment in a separate cohort of mice (cohort 3; n = 7 to 8). (f) Fed glucose was increased after 3 weeks of corticosterone treatment (n = 7 to 8). (g) Genes associated with gluconeogenesis were not increased after 2 days (n = 7 to 8). (h) However, these genes were increased after 3 weeks of corticosterone treatment (n = 7 to 8). White bars indicate vehicle; black bars, 75 µg/mL corticosterone treatment. *P < 0.05; **P < 0.01; ***P < 0.001. (a) Analyzed by 4PL data logged to normalize two-way ANOVA with Fisher least square difference post-test; (b, c, g, h) unpaired Mann-Whitney *t* test; (e) unpaired Student *t* test; and (f) two-way ANOVA with Fisher least square difference post-test.

Circulating insulin was increased 10-fold after 1 day of corticosterone treatment, with a further increase observed after 3 weeks of treatment (Fig. 4a). Because of this rapid and substantial increase in circulating insulin, we studied the expression of a number of well-characterized genes recognized to correlate with insulin resistance. After only 2 days of corticosterone treatment, a decrease was found in *Irs1* in the skeletal muscle, liver, and epididymal fat tissue (Fig. 4b). PI3-kinase subunit *P85α* mRNA expression increased in skeletal muscle, liver, BAT, and inguinal fat pads (data not shown). In BAT, the mRNA expression of another PI3-kinase subunit, *P110β*, was decreased (data not shown), indicating that features of early insulin resistance might be present in all the tissues examined.

After 3 weeks of chronic corticosterone treatment, further changes were found in *Irs1*, *P85α*, and *P110β* mRNA in skeletal muscle, WAT, and BAT in a manner associated with insulin resistance (Fig. 4c; data not shown). After 3 weeks of corticosterone, clear hepatic steatosis was present, which had not been present at 2 days (Fig. 4d). In the liver, *Irs1* mRNA was decreased (Fig. 4c) and *P85α* was increased (data not shown). In keeping with systemic insulin resistance, after chronic corticosterone treatment, an increase was found in the homeostatic model assessment for insulin resistance (Fig. 4e).

Hyperglycemia was not present during the early stages of corticosterone treatment but had developed by 3 weeks in the corticosterone-treated mice (Fig. 4f). *G6pc* (glucose 6-phosphate), *Gck* (glucokinase), *Slc2a2* (GLUT-2), and *Pygl* (glycogen phosphorylase) expression in the liver were all unchanged by short-term corticosterone administration (Fig. 4g) but were increased after 3 weeks corticosterone treatment (Fig. 4h). *Pck1/2* (PEPCK) was not altered at either time (Fig. 4g and 4h).

### *Agrp* expression is elevated with corticosterone treatment

**Figure 5.**
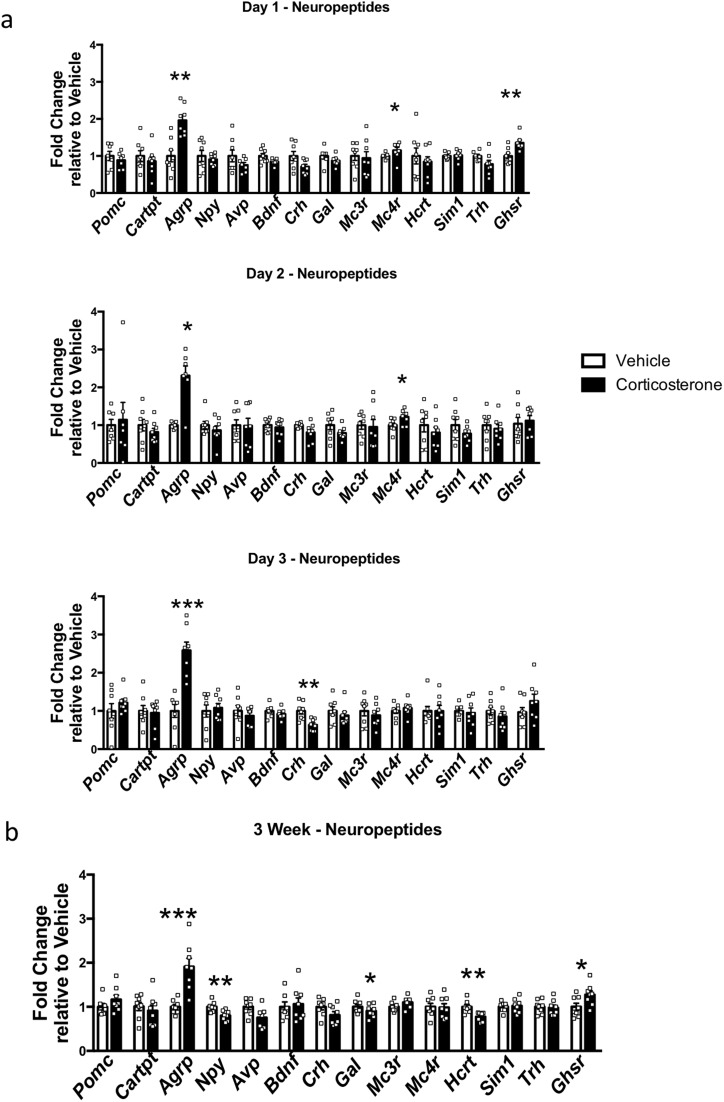
Corticosterone treatment increased *Agrp* mRNA expression. (a) Hypothalamic analysis of genes involved in energy balance after 1, 2, or 3 days or (b) 3 weeks of corticosterone treatment (n = 7 to 8). White bars indicate vehicle; black bars, 75 µg/mL corticosterone treatment. *P < 0.05; **P < 0.01; ***P < 0.001, unpaired Mann-Whitney *t* test.

Central hypothalamic mechanisms have an important role in the control of peripheral metabolism, and we have previously shown that corticosterone can increase *Agrp* after 4 weeks of corticosterone treatment. However, in the present study, we have extended our findings using groups of mice treated with corticosterone for 1, 2, and 3 days. Corticosterone treatment induced an increase in *Agrp* expression after only 1 day, which was sustained at days 2 and 3 (Fig. 5a). Furthermore, *Agrp* was still increased in the mice treated with corticosterone for 3 weeks (Fig. 5b). None of the other genes analyzed showed such a pronounced or persistent change in expression (Fig. 5a).

### Knockout of *Agrp* by CRISPR-Cas9

**Figure 6.**
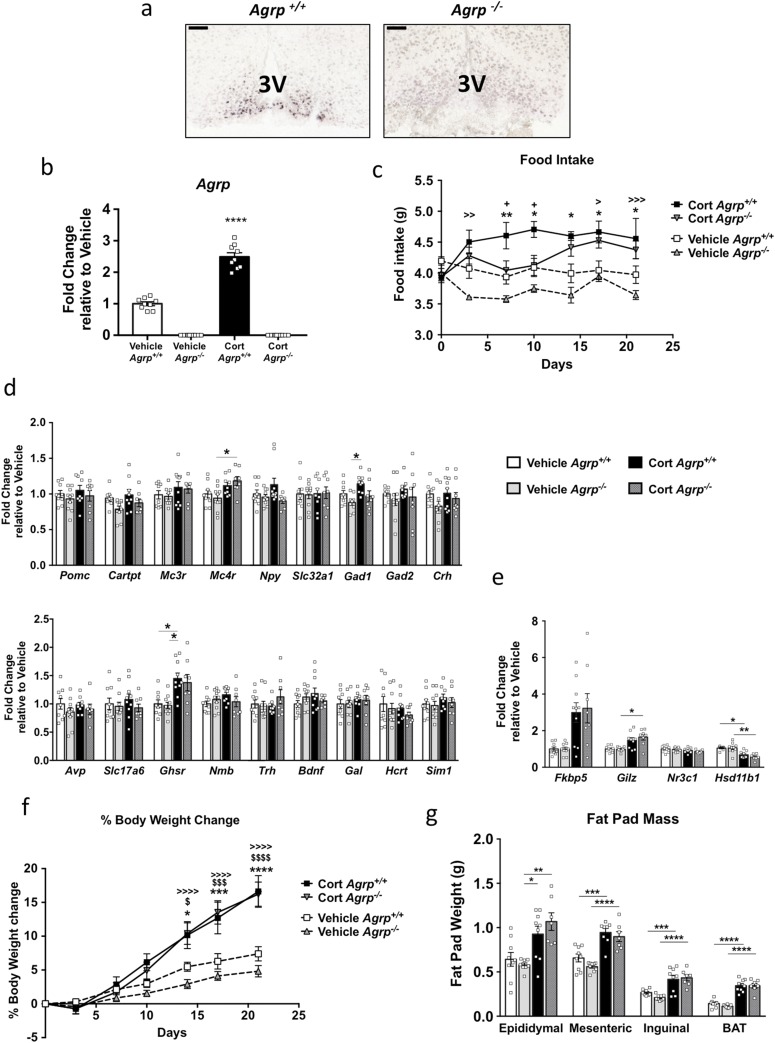
Loss of *Agrp* partially protected from corticosterone-induced hyperphagia. (a) Representative colorimetric *in situ* hybridization from *Agrp*^+/+^ and *Agrp*^−/−^ mice (n = 3). (b) *Agrp* mRNA was absent in *Agrp*^−/−^ mice (n = 9 to 10). (c) Twice-weekly food intake during 3 weeks of corticosterone treatment (n = 7 to 10). Hypothalamic analysis of genes involved in (d) energy balance (n = 7 to 10) and (e) glucocorticoid-responsive genes after 3 weeks of corticosterone treatment in *Agrp*^+/+^ and *Agrp*^−/−^ mice (n = 7 to 10). *P < 0.05; **P < 0.01; ***P < 0.001. (f) Percentage of body weight change during 3 weeks of corticosterone administration. (g) Epididymal, mesenteric, inguinal, and BAT mass after 3 weeks of corticosterone treatment. White bars and symbols indicate vehicle *Agrp*^+/+^; gray bars and symbols, vehicle *Agrp*^−/−^; black bars and symbols, 75 µg/mL corticosterone-treated *Agrp*^+/+^; gray-hatched and symbols, 75 µg/mL corticosterone-treated *Agrp*^−/−^. *Vehicle *Agrp*^+/+^ vs corticosterone *Agrp*^+/+^; >vehicle *Agrp*^−/−^ vs corticosterone *Agrp*^−/−^ ; +corticosterone *Agrp*^+/+^ vs corticosterone *Agrp*^−/−^. (b, d, e) Kruskal-Wallis *t* test with Dunn multiple comparison test; (c, f) two-way ANOVA, Tukey multiple comparison test; and (g) one-way ANOVA, Tukey multiple comparison.

To investigate whether the robust *Agrp* response to exogenous corticosterone was necessary for the development of the adverse effects observed, we studied the effect of corticosterone in a mouse engineered to lack *Agrp*. CRISPR-Cas9 was used to develop an *Agrp* null mouse (*Agrp*^−/−^). *In situ* hybridization confirmed the loss of *Agrp* in *Agrp*^−/−^ mice compared with littermate controls (*Agrp*^+/+^; Fig. 6a). In addition, real-time quantitative PCR confirmed the loss of *Agrp* in whole hypothalami from *Agrp*^−/−^ mice (Fig. 6b). As expected, *Agrp* was not detectable in the corticosterone-treated *Agrp*^−/−^ mice.

### Loss of *Agrp* partially protects male *Agrp*^−/−^ mice from corticosterone-induced hyperphagia but does not protect against body weight gain

As previously shown, *Agrp* expression was increased in corticosterone-treated *Agrp*^+/+^ mice (Fig. 6b). Loss of *Agrp* partially protected mice against corticosterone-induced hyperphagia (Fig. 6c). Analysis using two-way ANOVA indicated an interaction (*P* < 0.001) between genotype and corticosterone treatment, making the data more difficult to interpret. Therefore, a *post hoc* Tukey multiple comparison test was used and showed differences in corticosterone effects between corticosterone–*Agrp*^+/+^ and corticosterone–*Agrp*^−/−^ mice at days 7 and 10. Corticosterone induced a similar increase in food intake in *Agrp*^−/−^ compared with their *Agrp*^+/+^ littermates after day 10. To investigate whether other genes were driving the hyperphagia during this period, we performed mRNA analysis on a range of genes encoding neuropeptides and receptors involved in energy homeostasis. Loss of *Agrp* did not alter the basal expression of any of these genes (Fig. 6d). In the hypothalamus, glucocorticoid-responsive genes (*Fkbp5*, *Gilz*, *Nr3c1*, *Hsd11b1*) changed with corticosterone in both corticosterone–*Agrp*^−/−^ mice and corticosterone–*Agrp*^+/+^ mice, indicating that corticosterone was acting similarly in the hypothalamus of *Agrp*^−/−^ and *Agrp*^+/+^ mice (Fig. 6e).

Both *Agrp*^+/+^ and *Agrp*^−/−^ mice had a similar magnitude of increase in body weight with corticosterone treatment (Fig. 6f). At the end of the study, BAT, epididymal, mesenteric, and inguinal fat had increased to a similar extent in the corticosterone-treated *Agrp*^+/+^ and *Agrp*^−/−^ mice (Fig. 6g).

### *Agrp* deletion does not protect from corticosterone-induced hepatic steatosis, insulin resistance, or hyperglycemia

**Figure 7.**
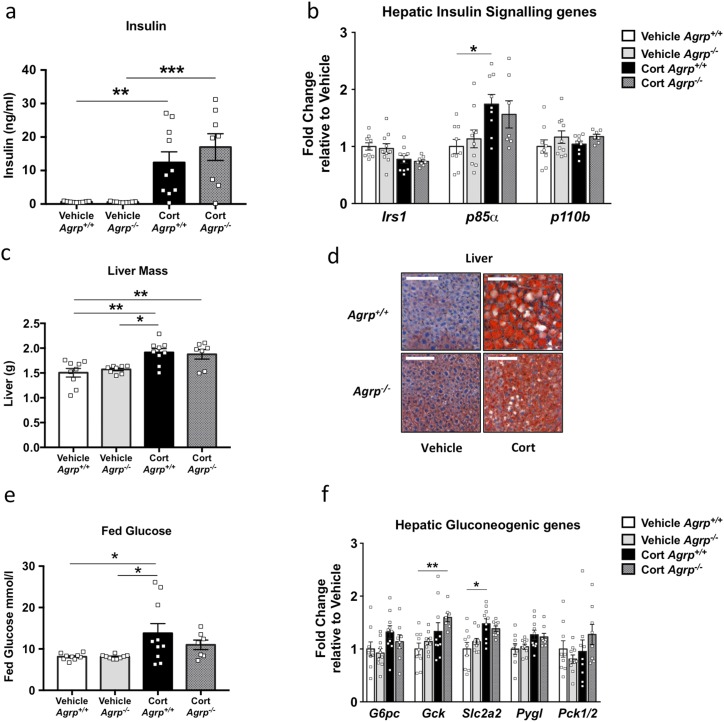
Loss of *Agrp* did not protect mice from corticosterone-induced insulin resistance or hyperglycemia. (a) Circulating insulin after 3 weeks of corticosterone treatment was increased in *Agrp*^+/+^ and *Agrp*^−/−^ mice (n = 8 to 10). (b) Hepatic *Irs1*, *P85a*, and *P110b* mRNA expression (n = 8 to 10). (c) Liver mass was not different between *Agrp*^+/+^ and *Agrp*^−/−^ mice (n = 7 to 10). (d) Oil red O staining in liver after 3 weeks of corticosterone treatment. Scale bar, 100 µm (n = 3 to 4; six to nine sections per mouse). (e) Fed glucose levels (n = 7 to 10) and (f) hepatic gluconeogenic genes (n = 8 to 10) in *Agrp*^+/+^ and *Agrp*^−/−^ mice. *P < 0.05; **P < 0.01; ***P < 0.001. (a) 4PL analysis, one-way ANOVA, Tukey multiple comparison; (c, e) one-way ANOVA, Tukey multiple comparison; and (b, f) Kruskal-Wallis *t* test with Dunn multiple comparison test.

After 3 weeks corticosterone treatment, hyperinsulinemia was present in both *Agrp*^+/+^ and *Agrp^−/−^* mice (Fig. 7a). In addition, quantification of a number of well-characterized hepatic insulin signaling genes (*Irs1*, *P85a*, and *P110b*) that have been recognized to correlate with insulin resistance indicated that loss of *Agrp* did not prevent the changes in these genes (Fig. 7b). The liver mass increased in both *Agrp*^+/+^ and *Agrp*^−/−^ mice with chronic corticosterone treatment (Fig. 7c), and this had resulted from increased lipid deposition (Fig. 7d).

Hyperglycemia developed in the corticosterone–*Agrp*^+/+^ mice after 3 weeks, and a nonsignificant trend was found toward an increase in blood glucose levels in the *Agrp*^−/−^ mice (Fig. 7e). In both *Agrp*^+/+^ and *Agrp*^−/−^ corticosterone-treated mice, evidence was found of changes in the genes involved in gluconeogenesis, even in the fed state (Fig. 7f).

## Discussion

The present study of chronic corticosterone treatment in mice identified a complex array of metabolic changes that evolved over time and involved both peripheral and central changes in pathways regulating energy balance. With corticosterone treatment, a rapid increase occurred in food intake, which was sustained for 3 weeks. Corticosterone treatment caused an increase in body weight, although not until day 10, and this change was not associated with altered energy expenditure. Corticosterone treatment also induced hyperinsulinemia after 1 day, with early changes in the expression of genes associated with insulin resistance in several peripheral tissues. This subsequently resulted in hyperglycemia at 3 weeks.

Our model of chronic corticosterone treatment supports and extends the results of other elegant studies using similar approaches but giving greater doses of corticosterone (1, 28–30), indicating that this is a reliable approach for addressing the different potential mechanisms underlying glucocorticoid-induced metabolic abnormalities. Mice given corticosterone, when glucocorticoid receptor is knocked down in adipose tissue, have improved glucose tolerance and insulin sensitivity (29). Other studies, in which mice had been given corticosterone when *Hsd11b1* (the enzyme responsible for the regeneration of corticosterone) had been deleted, had identified glucocorticoid regeneration in adipose tissue as a key factor in corticosterone-induced hepatic steatosis and fatty acid excess (28). Using a different approach, Bowles *et al.* (30) found that endocannabinoid signaling in the liver was necessary to mediate the adverse effects of corticosterone on hepatic steatosis and dyslipidemia.

One of the most striking findings of the present study was the marked effect of chronic corticosterone treatment in increasing food intake and hypothalamic *Agrp* throughout the study period, with no consistent change in any of the genes encoding signaling molecules and receptors known to be involved in energy balance. The ability of glucocorticoids to increase *Agrp* expression is supported by our previous studies (3, 6). Furthermore, considerable evidence has shown that AgRP promotes hyperphagia (31, 32); therefore, the increase in *Agrp* would be predicted to contribute to the increase in food intake observed.

To test whether the AgRP neuropeptide mediated the corticosterone-induced hyperphagia, we developed mice with ablation of the *Agrp* gene. When these mice were treated with chronic corticosterone, an initial phase occurred in which food intake was not increased compared with corticosterone-treated *Agrp*^+/+^ mice. However, we did not observe the expected protection from corticosterone-induced hyperphagia after day 10. This was surprising given the markedly increased *Agrp* expression in wild-type mice treated with corticosterone, which was consistent for several groups of mice. However, we acknowledge that factors, other than those we analyzed in the present study, might have played a part in glucocorticoid-induced hyperphagia.

Although food intake increased after 1 day corticosterone treatment, the body weight did not increase until day 10, which suggests that corticosterone might also be affecting energy expenditure. Some of our data indicated that corticosterone treatment might be reducing the metabolic activity of BAT, both in the early stages and at the end of the study. Owing to the increased lipid deposition, BAT weight was increased from day 1, which has been previously reported to be indicative of less-active BAT (33). Furthermore, with corticosterone treatment, the protein levels of UCP-1 and the mRNA expression of *Prdm16*, *Ucp1*, *Cidea*, and *Ppargc* in BAT had all decreased. These are all markers that support the possibility of decreased energy expenditure (34–36). However, we were unable to identify any differences in energy expenditure between the corticosterone and vehicle group, either before or after the body weight gain. Nevertheless, with corticosterone treatment, we did observe a sustained elevation in RER during the inactive phase at either the earlier evaluation point or at day 14, potentially indicating a change in nutrient partitioning and decreased metabolism of lipids.

Glucocorticoids are known to act directly on BAT (37, 38). However, the effects of glucocorticoids on BAT, in the present study, were similar to those seen in mice with antagonism of the MC4R by SHU9119, suggesting that glucocorticoid-induced AgRP antagonism of MC4R might be causing these similar effects. In studies by Kooijman *et al.* (39), using SHU9119, a decrease in melanocortin tone in the hypothalamus led to lipid infiltration and a rapid increase in BAT mass, a decrease in UCP-1 protein expression, an increase in RER, and decrease in energy expenditure. A second study using SHU9119 to chronically blockade central melanocortin receptors did not report a change in energy expenditure but did find an elevation in RER and reduced *Ucp1* expression in BAT (40). However, in our study, no difference was found in BAT weight between *Agrp*^+/+^ and *Agrp*^−/−^ mice treated with corticosterone, indicating that the elevation in *Agrp* with glucocorticoid treatment is not directly responsible for the elevation in BAT weight.

After 1 day of glucocorticoid treatment, the circulating insulin levels were increased, and after 2 days, changes were found in insulin signaling genes in skeletal muscle, WAT, liver, and BAT. Whether these changes had resulted from glucocorticoids acting centrally or peripherally is unknown. Deletion of glucocorticoid receptor in adipose tissue in adult mice has highlighted its importance in glucocorticoid-induced systemic insulin resistance (29). However, central mechanisms have also been proposed to cause peripheral insulin resistance. One mechanism includes AgRP neurons (16, 41), which also have a role in the control of hepatic glucose production (17). However, in the present study, removal of the AgRP peptide did not reverse the hyperinsulinemia or the effects on insulin signaling genes caused by chronic corticosterone. Furthermore, in the *Agrp*^−/−^ mice, there did not appear to be any protection from the corticosterone-induced changes observed in hepatic gluconeogenic genes. Therefore, AgRP is unlikely to mediate these glucocorticoid effects, although the present study only focused on measurement of insulin and genes previously shown to be modified in insulin-resistant states.

The present study also demonstrated that corticosterone treatment induced hyperglycemia, with blood glucose levels elevated between days 17 and 21. In the early phase (day 2 of corticosterone treatment), rapid induction of hyperinsulinemia likely prevented the early development of hyperglycemia. This elevation in circulating insulin might also have counteracted the ability of glucocorticoids to directly affect the expression of *Pck1/2* or *G6pc* in the liver (42). Additionally, we observed elevated circulating leptin, which, through a central action, might have diminished hepatic gluconeogenesis (43).

In conclusion, in this model of glucocorticoid treatment, there was an evolution of changing metabolic abnormalities, which finally led to the weight gain and hyperglycemia that characterize the metabolic adverse effects of glucocorticoid treatment in humans. Our study investigated whether chronic corticosterone treatment targets the hypothalamic pathways involved in energy balance. However, despite a large and consistent increase in hypothalamic *Agrp*, it appears that this neuropeptide does not have a central role in glucocorticoid-mediated metabolic adverse events.

## Abbreviations:

ANCOVA: analysis of covariance
BAT: brown adipose tissue
CRISPR: clustered regularly interspaced short palindromic repeats
sgRNA: single-guide RNA
TBS-T: Tris-buffered saline-Tween 20
WAT: white adipose tissue
